# Lumbar lordosis and sacral slope do not differ in two types of postoperative lumbar disc re-herniation: a cross-sectional observational study

**DOI:** 10.1186/s12891-024-07376-3

**Published:** 2024-04-06

**Authors:** Zhijia Shen, Wenhao Wang, Li Ni, Hongcheng Zhao, Lianda Yang, Huilin Yang, Linlin Zhang

**Affiliations:** 1 Department of Surgery, The First Affiliated Hospital of Soochow University, Suzhou, Jiangsu 215006 China; 2 Suzhou Medical College of Soochow University, 199 Ren’ai Road, Suzhou, Jiangsu 215123 China

**Keywords:** Lumbar disc re-herniation, Spinal sagittal parameters, Lumbar lordosis, Sacral slope

## Abstract

**Background:**

To identify the differences of lumbar lordosis (LL) and sacral slope (SS) angles between two types of postoperative lumbar disc re-herniation, including the recurrence of same level and adjacent segment herniation (ASH).

**Methods:**

We searched the medical records of lumbar disc herniation (LDH) patients with re-herniation with complete imaging data (*n* = 58) from January 1, 2013 to December 30, 2020 in our hospital. After matching for age and sex, 58 patients with LDH without re-herniation from the same period operated by the same treatment group in our hospital were served as a control group. Re-herniation patients were divided into two groups, same-level recurrent lumbar disc herniation group (rLDHG) and adjacent segment herniation group with or without recurrence (ASHG). The preoperative, postoperative and one month after operation LL and SS were measured on standing radiographs and compared with the control group by using t-test, ANOVA, and rank-sum test. Next, we calculated the odds ratios (ORs) by unconditional logistic regression, progressively adjusted for other confounding factors.

**Results:**

Compared with the control group, the postoperative LL and SS were significantly lower in LDH patients with re-herniation. However, there were no differences in LL and SS between ASHG and rLDHG at any stage. After progressive adjustment for confounding factors, no matter what stage is, LL and SS remained unassociated with the two types of re-herniation.

**Conclusions:**

Low postoperative LL and SS angles are associated with degeneration of the remaining disc. Low LL and SS may be independent risk factors for re-herniation but cannot determine type of recurrence (same or adjacent disc level).

## Background

Lumbar discectomy is beneficial for patients with lumbar disc herniation (LDH) [1, 2]. However, recurrent lumbar disc herniation (rLDH) is one of the important reasons for unsatisfactory results and reported in 5–21%, with an increasing incidence over time. Moreover adjacent segment herniation (ASH) is another cause [1–11]. In addition to exacerbating already uncomfortable symptoms, rLDH and ASH may also necessitate a secondary operation, which is usually more complicated than the initial intervention because of postoperative adhesions.

Various risk factors for rLDH and ASH have been investigated in recent decades, mainly focused on population characteristics, like age, smoking status, gander, body mass index (BMI), and certain some radiologically identifiable factors, such as disc degeneration, disc height, and sagittal range of motion (sROM) [2, 4–8, 10, 12–17]. However, few studies have focused on the relationship between lumbosacral parameters including lumbar lordosis (LL) and sacral slope (SS) and re-herniation. LL and SS have been considered possible risk factors of ASH in some studies [10, 18], but it is still unclear whether LL and SS have an effect on rLDH. Further, the existence of similar imbalances in rLDH and ASH patients is worthy of further investigation.

In this study, we attempted to compare the LL and SS angles that are easily calculated from radiographs to identify the difference between patients with re-herniation and LDH patients without re-herniation. We further compared LL and SS in patients with rLDH and ASH, to our knowledge, has not been done before. Given the significant effect of biomechanics in LDH, we believe that LL and SS could be risk factors for re-herniation and may be different in the two types of re-herniation. We believe our results are important as it allows clinicians and researchers to explore the role of the two lumbosacral sagittal parameters in predicting recurrence or ASH, thereby providing hints and references for the future prediction models. An excellent model to predict two types of re-herniation is necessary, in which sagittal balance is an important factor, and it is unreasonable not to mention it.

## Methods

### Patients

This was an observational clinical study. We collected the data of all patients who meet our inclusion criteria. In all, 58 patients who had re-herniation with complete imaging data from January 1, 2013 to December 30, 2020 in our hospital were included, consisting in group B. Of these, 42 (72.4%) patients were male and the mean age was 50.59 ± 12.42 years (range, 25–72 years). We collected all the patients who experienced initial lumbar disc operation in a same treatment team in our hospital from 2013 to 2020, then we contacted them by phone or e-mail to make sure whether they were experiencing recurrent leg pain. Anyone who had symptom should provide their latest lumbar MRI in or out of our hospital, and the MRI was interpreted by doctors in our radiology department who were blinded to the patients’ condition and the experiment and further reviewed by two senior orthopedic surgeons. Finally, all the patients with re-herniation who agreed with the study were included. To reduce selection bias, we collected the data of all LDH people without re-herniation after matching for age and sex during the same stage, served as control group (group A). The control group (*n* = 58) was randomly selected from a cohort of age- and sex-matched candidates.

Recurrent LDH can be defined as disc herniation at the same level, regardless of ipsilateral or contralateral herniation, in a patient who experienced a pain-free interval of at least 6 months after surgery [4]. Although, ASH is generally considered as re-herniation at the adjacent segment to the initial hernia site after discectomy, its definition is yet not clear. Besides, ASH can include with or without recurrence in initial segment, so we had to adjust our criteria and groupings. Hence, patients included in this study were required to meet the following strict inclusion criteria: [1] LDH with clinical symptoms and clear diagnosis, and primary discectomy [2], absence of leg pain for at least 6 months after the primary operation, and [3] follow-up MRI showing new disc re-herniation at the previously operated level or adjacent segment, either ipsilateral- or contralateral. Patients with history of any other spinal surgery except discectomy or any other spinal disease, such as spinal deformity, spine fracture and infection, lumbar spondylolisthesis and degenerative stenosis of the spinal canal were excluded. We set up objective criteria and included every patient meeting those criteria to avoid subjective bias. The study patients were divided into two groups according to recurrence and ASH, namely, same-level recurrent lumbar disc herniation group (rLDHG) and the adjacent segment herniation group with or without recurrence at the initial level (ASHG).

The surgery procedures included traditional solely discectomy by micro-endoscopy or open discectomy, and discectomy by spinal endoscopy through transforaminal approach (above the L4–L5 level), or interlaminar approach (at the L5–S1 level). Primary surgery was performed in our hospital and by a same treatment group, and all the surgeries were performed by expert surgeons with extensive experience in discectomy to confirm that all enrolled patients had undergone the above surgeries by same surgical procedures.

The re-herniation interval was calculated from the day of the first surgery to the date that rLDH or ASH was diagnosed.

### Definition of comorbidities and confounding factors

In our study, we also take some confounding factors into consideration. Age and sex were obtained in medical record. In view of the inaccuracy of the medical records initially recorded, we asked enrolled patients about their comorbidities at return visits. The definition for smoking and alcohol use: smoked at least 100 cigarettes in life and had at least 12 alcohol drinks in the past one year, and an alcohol drink equals to 12 oz. beer, a 5 oz. glass of wine, or one and a half ounces of liquor. Hypertension and Diabetes Mellitus were defined as having diagnosed of Hypertension and Diabetes Mellitus by doctors or taking medicine treating the disease.

### Radiological measurement

**Fig. 1:**
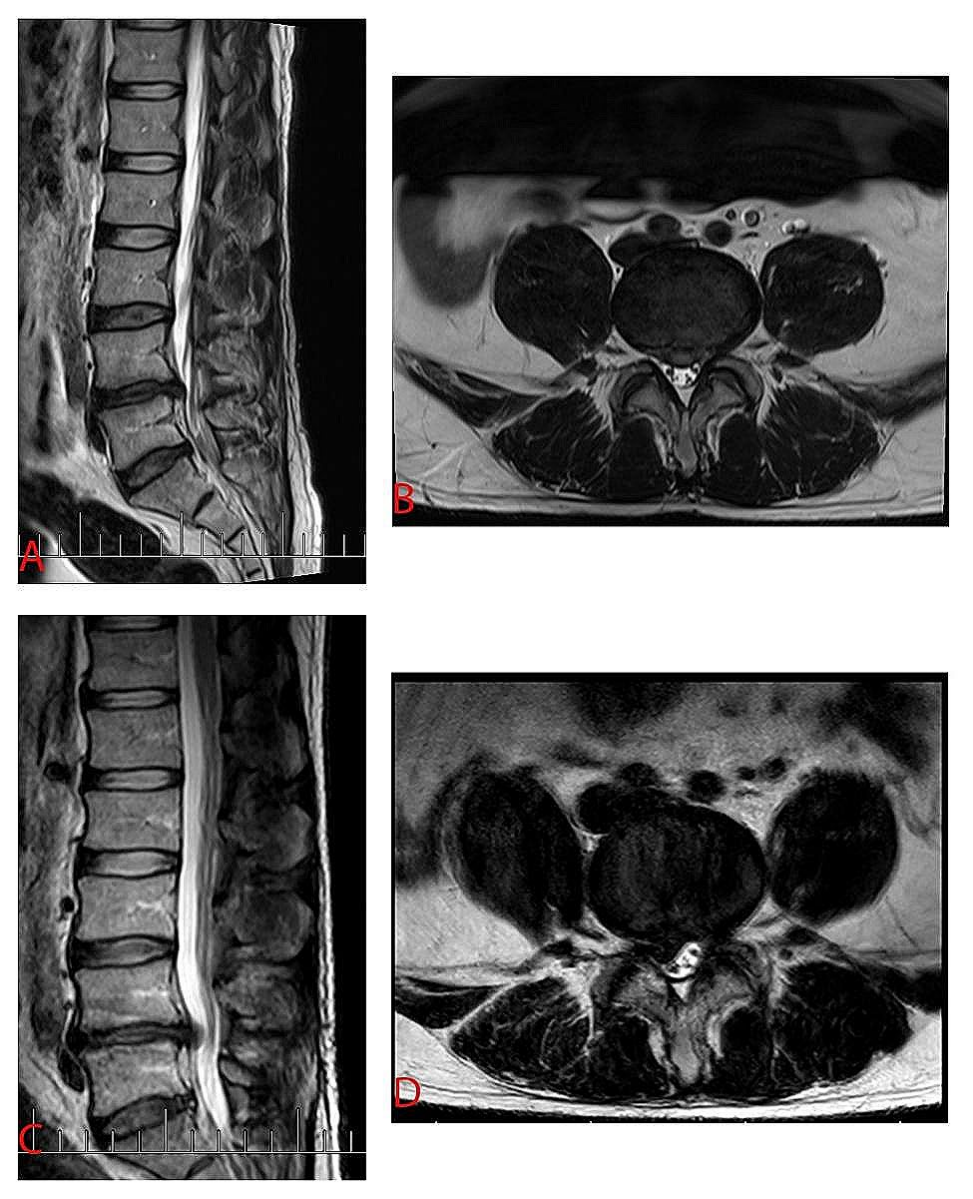
MRI images shows LDH and re-herniation in the patient. *Note*. **(A)** T2-weighted sagittal lumbar MRI shows LDH at L4-L5 segment. **(B)** Cross section of L4-L5 segment on T2-weighted MRI shows LDH. **(C)** T2-weighted sagittal lumbar MRI shows re-herniation at L4-L5 segment after discectomy by spinal endoscopy through the transforaminal approach. **(D)** Cross section of L4-L5 segment on T2-weighted MRI shows re-herniation

**Fig. 2:**
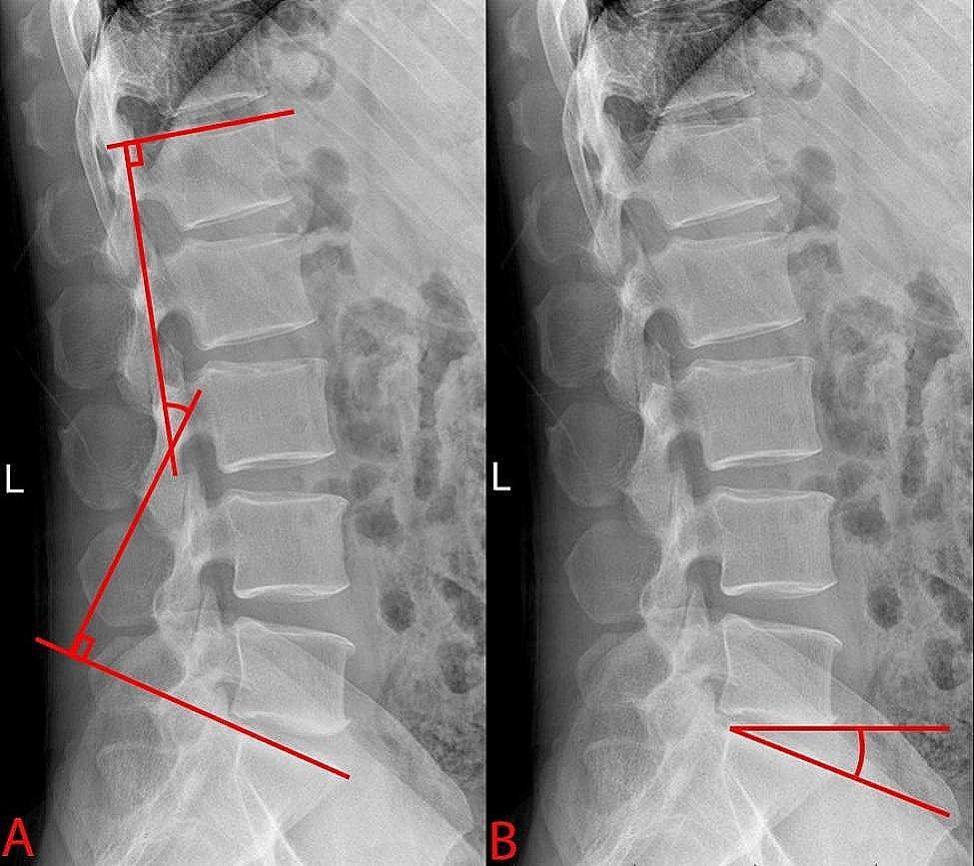
The measurement method of lumbar lordosis angle and sacral slop angle. *Note*. Standing radiograph shows lumbar lordosis angle **(A)** and sacral slope angle **(B)**

MRI was recommended for all postoperative patients experiencing recurrent leg pain, because MRI is essential to confirm the presence or absence of rLDH and ASH (Fig. 1). MRI could show the protruding nucleus pulposus and the degree of nerve compression clearly. The postoperative MRI was interpreted by doctors in our radiology department who were blinded to the patients’ condition and the experiment and further reviewed by two senior orthopedic surgeons. On the other hand, sagittal parameters should be measured at standing radiographs, because X-rays show bone structures more clearly, and make it easy to measure angles. Many studies have proven that LL and SS at standing radiographs may significantly higher than those at MRI, and standing position might be more in line with the way humans move [19, 20]. So, we choose to measure angles at standing radiographs. An experienced senior investigator measured the LL and SS on standing radiographs at preoperation, postoperation and one month after operation (for easy expression, PreLL, PreSS, PostLL, PostSS, one mLL and one mSS, respectively), the measurements were taken in duplicate and the average result was considered. As shown in Fig. 2, LL is the lordotic angle of the lumbar; it is the angle between the parallel line from the upper end plate of the L1 vertebrae and the line from the upper final plate of the sacral vertebrae in the sagittal plane. The angle of SS was the angle between the parallel line passing through the upper plate of the sacrum and the horizontal line.

### Statistical analysis

Statistical analysis was performed using SPSS software for Windows (version 26.0; IBM, Armonk, NY, USA). The mean or median values of LL and SS of group B were compared with those of group A or between rLDHG and ASHG by t-test, ANOVA, and the Kruskal-Wallis H test. Mean and standard deviation for variables with normal distribution were used to express descriptive statistics. Next, we calculated odds ratios (ORs) for the risk of re-herniation compared with the control group or rLDHG compared with ASHG by unconditional logistic regression. For all analyses, *P* ≤ 0.05 was accepted as statistically significant.

## Results

### Group A vs. group B

As shown in Table 1, we confirmed that smoking and alcohol drinking significantly affect the lumbar disc re-herniation after surgery (*p* = 0.025, *p* = 0.016, respectively). BMI, hypertension and diabetes mellitus have no differences in the two groups (*p* = 0.590, *p* = 0.552, *p* = 0.284, respectively).

**Table 1:** Patients demographics

Parameter		Group A(*n* = 58)	Group B (*n* = 58)	P value
Age (yrs)	Mean (SD)	50.59(12.42)	50.59(12.42)	/
	Range	25–72	25–72	
Gender	Female(n%)	16(27.6%)	16(27.6%)	/
	Male(n%)	42(72.4%)	42(72.4%)	
BMI	Mean(SD)	24.83(2.91)	25.14(3.12)	0.590
	Range	22.20-31.51	19.15–32.19	
Comorbidty(n)				
Hypertension		20(34.48%)	17(29.31%)	0.552
Diabetes mellitus		6(10.34%)	10(17.24%)	0.284
Smoking		5(8.62%)	14(24.14%)	0.025*
Alcohol drinking		1(1.72%)	8(13.79%)	0.016*

BMI, body mass index;

*  Significance between the two groups, P<0.05.

As shown in Table 2, postLL, postSS and one mSS of group A were significantly higher than those of group B (*p* = 0.049, *p* = 0.022, *p* = 0.038, respectively). PreLL, preSS and one mLL were higher in group A, however, there were no statistical significance (*p* = 0.058, *p* = 0.118, *p* = 0.159, respectively).

**Table 2:** Spino-peivic sagittal balance parameters of both groups

	Group A(*n* = 58)	Group B(*n* = 58)	P-Value
PRE			
LL(°)	37.74 ± 14.27	33.02 ± 12.18	0.058
SS(°)	30.59 ± 11.56	27.66 ± 8.19	0.118
POST			
LL(°)	40.14 ± 11.01	36.03 ± 11.19	0.049*
SS(°)	32.76 ± 8.91	29.05 ± 8.26	0.022*
ONE M			
LL(°)	39.83 ± 11.61	36.81 ± 11.29	0.159
SS(°)	31.16 ± 8.08	28.12 ± 7.45	0.038*

LL, lumbar lordosis; SS, sacral slope; PRE, preoperative; POST, post-operative; ONE M, one month after surgery

*Significance between the two groups, *P* < 0.05

**Fig. 3:**
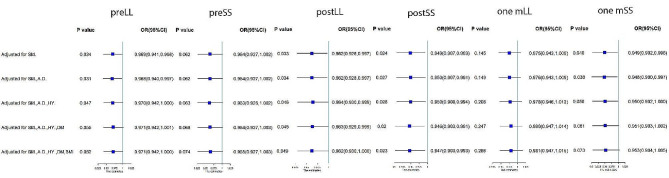
The result of unconditional logistic regression, progressively adjusted for smoking (SM.), alcohol drinking (A.D.), hypertension (HY.), diabetes mellitus (DM) and body mass index (BMI)

Further, in order to verify the robust of our results, we calculated odds ratios (ORs) of every angle by progressive logistics regression, adjusted for smoking, alcohol drinking, hypertension and other confounding factors (Fig. 3). PostLL and postSS were significantly higher in group A than those of group B in final model, ORs were 0.962 (0.930-1.000) and 0.947 (0.903–0.993). However, one mSS lost statistical significance after adjustment for all confounding factors. (*p* = 0.049, *p* = 0.023, *p* = 0.073, respectively). Compared to control group, the ORs of LDH patients with re-herniation of the three stages LL or SS and its 95% CI was shown in the Fig. 3.

### rLDHG vs. ASHG

As shown in Table 3, according to rLDH and ASH, we divided the 58 subjects into two groups. ASHG had 25 patients (18[72.0%] male and 7[28.0%] female; mean age: 54.60 ± 11.91 years; age range: 46–72 years). rLDHG had 33 patients (24[72.7%] male and 9[27.3%] females; mean age: 47.55 ± 12.10 years; age range: 25–71 years). Patients in ASHG were significantly older than those in rLDHG (*p* = 0.031).

**Table 3:** Demographic data of ASHG and rLDHG

Parameter		rLDHG(*n* = 33)	ASHG(*n* = 25)	P value
Age (yrs)	Mean (SD)	47.55(12.10)	54.60(11.91)	0.031*
	Range	25–71	46–72	
Gender	Female(n%)	9(27.3%)	7(28.0%)	0.951
	Male(n%)	24(72.7%)	18(72.0%)	
BMI	Mean(SD)	25.04(3.23)	25.26(3.02)	0.793
	Range	19.15–29.68	25.08–32.19	
Comorbidty(n)				
Hypertension		9(27.27%)	8(32.00%)	0.698
Diabetes mellitus		5(15.15%)	5(20.00%)	0.631
Smoking		3(9.09%)	11(44.00%)	0.002**
Alcohol drinking		2(6.06%)	6(24.00%)	0.052

BMI, body mass index;

* Significance between the two groups, P<0.05; ** Significance between the two groups, P<0.01

**Fig. 4:**
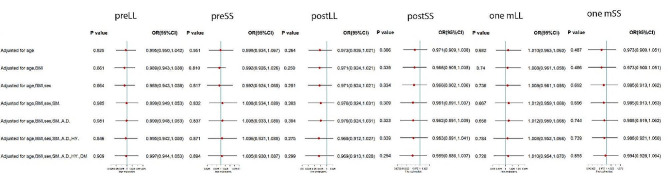
The forest plot of OR and its 95%CI of ASH of the three stages LL and SS in unconditional logistic regression, compared to rLDH, progressively adjusted for sex, age, smoking (SM.), alcohol drinking (A.D.), hypertension (HY.), diabetes mellitus (DM) and body mass index (BMI)

The three stages mean LL and SS angles of rLDHG and ASHG were presented in Table 4. However, there were no significant differences of LL and SS in the two types of re-herniation. After progressive adjustment for age, sex and other confounding factors, the three stages LL and SS remained no association with two types of re-herniation (Fig. 4).

**Table 4:** Spino-peivic sagittal balance parameters of ASHG and rLDHG

	rLDHG(*n* = 25)	ASHG(*n* = 33)	P-Value
LL(°)			
PRE	34.48 ± 11.97	31.91 ± 12.41	0.431
POST	38.20 ± 9.52	34.39 ± 12.19	0.202
ONE M	36.16 ± 9.77	37.30 ± 12.45	0.706
SS(°)			
PRE	28.04 ± 6.86	27.36 ± 9.16	0.758
POST	30.28 ± 5.53	28.12 ± 9.83	0.294
ONE M	28.40 ± 5.64	27.91 ± 8.56	0.795

LL, lumbar lordosis; SS, sacral slope; PRE, preoperative; POST, post-operative; ONE M, one month after surgery

*Significance between the two groups, *P* < 0.05

Overall, our results indicated that patients with lumbar disc re-herniation had lower postLL and postSS and one mSS than control group. But three stages LL and SS showed no differences between rLDHG and ASHG. Adjusted for all confounding factors, LL and SS remained no differences between the two types of re-herniation.

## Discussion

Previous large population-based studies have reported the risk of reoperation for LDH to be 10-15% [21–23]. rLDH and ASH have become increasingly common among patients who have undergone lumbar discectomy or fusion. Studies have suggested that male sex, being tall, indulging in heavy labor, and smoking may be predictors of rLDH [2, 4–8, 12–15]. In addition, BMI, age, length of fusion, smoking and sagittal alignment are considered possible risk factors of ASH [10, 18, 24, 25].

Studies on LDH have shown that low LL and SS angles cause the sagittal vertebral axis (SVA) to move forward such that the compressive stress and sheer force of the intervertebral disc increase with increased flexion activity [26]. These changes increase the compressive forces created by gravity and meanwhile, the absorption of the shaking loads formed by these vertical forces decrease. Therefore, low LL and SS can accelerate disc degeneration and cause stress occlusion, and this phenomenon results in an excess load on joints, muscles and ligaments around the spine, which greatly reduces the stability of the patient’s lumbar spine and accelerates the disc degeneration in turn [27]. Disc degeneration and mechanical instability are a vicious cycle that eventually results in LDH.

However, there were not enough articles focusing on the relationship between LL and SS and re-herniation. We explored the differences between LL and SS in patients with different types of re-herniation, which was innovative to an extent. Low LL and SS angles are clearly related to LDH, but their relationship with re-herniation is controversial, and to the best of our knowledge, the role between different types of re-herniation has not yet been explored before.

In our work, postLL and postSS in patients with re-herniation were statistically lower than in the control group. Some studies have found that compared to the LDH group, LL and SS angles were lower in patients with re-herniation and may increase the risk of lumbar disc degeneration and hence relate to re-herniation [10, 26, 28–31]. Furthermore, Belykh et al. [1]found that decreased LL angle was one of risk factors of rLDH. Kong et al. [32] suggested that small LL angle was an independent significant risk factor for rLDH after Percutaneous endoscopic lumbar discectomy (PETD). Wang et al. [10] in his meta-analysis found that low post-operative LL was associated with ASH. Taken together, the above findings show that low LL and SS angles do play a significant role in re-herniation. In Matsumoto and Ankrah’s studies [30, 31], there was no statistically significant difference in the pre- and post-operative LL and SS. Hence, as the operation accelerates disc degeneration after discectomy [11], low LL and SS angles which still exist after surgery accelerate the remaining disc degeneration, unidirectionally increasing the forward spinal pressure and causing the SVA to move further forward, thus disrupts the equilibrium of shear force and causes the shear force acting on the lumbar intervertebral disc to be more concentrated [33], weakens intervertebral space stability, and increases the retrusive trend of the residual intervertebral disc. As long as the above-mentioned sagittal unstable factors exist and continue to develop after operation, patients with low LL and SS still have a higher risk of rLDH than patients with normal LL and SS. A similar biomechanical mechanism also exists for ASH. Low LL and SS after operation not only influence the residual disc, but also accelerate the adjacent segment disc, eventually leading to ASH [28, 29, 31]. Recent studies have reported that failure of reestablishment of LL after discectomy seems to be a risk factor for ASH [34, 35], and thus the PI-LL (PI refers to pelvic incidence angle, which is a constant for a patient.) should appropriately match during surgery to prevent adjacent segment herniation [28]. Senteler et al. [36] found that a PI-LL mismatch ≥ 15° tends to predict an increased joint load at the segment close to the fusion level. Patients with low LL tend to be at risk of developing ASH because preoperative high PI-LL mismatch following the failure of rebuilding the LL angle would maintain or even increase. Therefore, the reestablishment of the normal relationship between LL and PI may be valuable for prognosis, and low LL and SS could increase the risk of ASH.

At the same time, another attempt in this study was that we compared LL and SS between rLDH and ASH, but no significant differences could be found. We believe that LL and SS in ASHG should been lower than patients in rLDHG. As is known, ASH may be induced by abnormal intradiscal pressure and too much movement at the adjacent segments [10]. Lower LL and SS may cause more severe degeneration to adjacent segments before the initial operation and may lead to more movement in the adjacent segment after discectomy. As a result, the compressive stress and sheer force of the intervertebral disc increases, causing further adjacent segment degeneration (ASD). Although the initial disc goes through double whammy of accelerating disc degeneration by operation and low LL and SS, it is a long process for it to herniate again. Thus, when adjacent segments degenerate to a critical state before operation and post-operative ASD has been worse than the initial disc degeneration, ASH may take place before rLDH. But the truth was disagreement with our conjecture. A possible explanation for this is as follows. In general, the degeneration of initial disc tends to be accelerated by surgical interfere, as a result of endplate degeneration and disc dehydration [11, 37], and is represented by a loss of disc height. Yorimitsu et al. found a 25% loss of disc height in most patients one year after a lumbar discectomy [38]. However, Axelsson and Karlsson [39] reported that the restabilization stage begins when the disc height is reduced by 50%. Besides, Kim et al. [4] suggested that collapsed discs are more stable than those with preserved disc height. However, a stable segment is associated with immobilization of the regarding segment which might induce an overload on the adjacent segments. [11] Dalgic et al. [11] think that After discectomy, collapsed discs are biomechanically more stable than those with preserved disc heights, and responses to axial compression on intervertebral disc pressure produced deformations of adjacent levels despite limitations. Thus, rLDH may took place before the re-stabilization of the primary disc, but ASD may further accelerate after the re-stabilization. Hence, we believe that LL and SS could affect the entire lumbar spine, but in partial segments, degeneration together with aging may be more significant in the terms of inducing re-herniation. Based on our research, postoperative rLDH and ASH are mainly decided by other factors. Similar to our results, Dalgic et al. [11] found that the mean age of ASHG was significantly higher than those with rLDHG. We believed that ASH patients might have gone through the instability phase of initial disc. The restabilized disc might accelerate ASD, with aging, and result in ASH in the end. So, the mean age of ASH patients was higher than that with rLDH. In two meta-analyses, Wang and Huang suggested that disc protrusion and diabetes were predictors for rLDH; body mass index (BMI), hypertension, post-operative LL, and preoperative PI were associated with the development of ASD; and smoking was a risk factor for both rLDH and ASD [8, 10]. Besides studies suggest that ASD and rLDH were the result of natural degeneration and have nothing to do with mechanics [40, 41]. Furthermore, recent studies suggest that there is an association between genetic influences and disc degeneration. Eser et al. believed that short repeated alleles of the aggrecan gene were significantly associated with disc degeneration and multilevel disc degeneration. Both genetic and environmental factors have been reported to influence lumbar disc re-herniation. LL and SS in patients were lower than those in the control population, suggesting that sagittal alignment might affect the occurrence of re-herniation, but LL and SS did not seem to determine which type of re-herniation can occur. Discovering why LL and SS accelerate re-herniation but cannot determine which type occurs is a possible promising future research direction.

We believe our study has clinical merit. PostLL and postSS are likely associated with re-herniation, although they do not affect the type of re-herniation. Our study may play a role in management for individuals before, after and during operation. For patients, we could set up a prediction model with LL and SS and other parameters to find individuals at risk of re-herniation, then we may take action to prevent re-herniation, including back extensor exercises. During operation, we should rebuild LL and SS angles properly, making PI-LL mismatch“15”to reduce the risk of re-herniation. Finally, for individuals after surgery, we could evaluate the operation effect by measuring the postoperative angles, and evaluate the risk of re-herniation every year by taking standing radiographs, in order to prevent re-herniation occurrence. In our perspective, our study might help a lot for patients at risk of re-herniation. besides above, more clinical applications are waiting for study. This highlights the importance of undertaking active measures to rebuild LL and SS to prevent re-herniation. So far, many approaches and interventions have been proved that they could help rebuild sagittal balance Clinicians could re-build LL and SS by proper pressure of pedicle screw, inserting a connecting rod bent according to the target curvature and wedge-shaped interbody fusion apparatus by operation. After operation, patients could take active back extensor exercises, delay loading, lose weight, avoid sitting or standing for long periods of time to prevent LL and SS reducing again. Besides, maintaining proper bone mineral density could also help maintain sagittal balance. However, regarding severe degeneration of adjacent segments before operation; it is not advisable to blindly pursue the recovery of the lumbosacral curve. It is necessary to reestablish the overall balance of lumbosacral region and control other possible risk factors to reduce the possibility of re-herniation in adjacent segments.

Our study has some limitations. First, the sample size of our study was small, due to the strict enrollment criteria, resulting in a small number of cases, and the reliability of the results needs to be proved by studies with larger sample sizes. Second, given the limitation of the range of lumbar MRI, we could only obtain LL and SS angles, and other sagittal parameters such as PI and PT (pelvic tilt) could not be measured. Therefore, our study was limited to just two parameters, and the relationship between other sagittal parameters and re-herniation could not be studied. EOS® imaging system has gain acceptance across the world because more and more applications of it are being discovered. Compared to X-rays, EOS not only has lower radiation dose, but also has ability to reconstruct three dimensional (3D) images and image the whole body including the spine and lower limbs in the functional standing position [42]. So, we could determine the alignment of the center points of both femoral heads, then we could measure other sagittal parameters, such as PI and PT. Third, as a cross-sectional observational study, the ability to control confounding bias was insufficient. Although we take some confounding factors into consideration, other factors, for example, the time between initial surgery and re-herniation occurrence, might influence these parameters. We plan to study the time between surgery and re-herniation that might influence the sagittal parameters and the type of re-herniation in the following study. Besides, future prospective studies are needed to further clarify the relationship between LL and SS and re-herniation. Finally, we only included patients with MR imaging data; this may have caused some selection bias.

## Conclusions

Low postoperative LL and SS angles are associated with degeneration of the remaining disc. Low LL and SS may be independent risk factors for re-herniation but cannot determine type of recurrence (same or adjacent disc level).

## Data Availability

The datasets generated and/or analyzed during the current study are not publicly available, but are available from the corresponding author on reasonable request. Our data contained private information such as patients’ names and hospital numbers, and some patients requested that their personal information not be disclosed directly unless they had personally confirmed it. Therefore, if you need relevant data, you can contact the corresponding author and obtain the consent of these patients before obtaining the data.

## Abbreviations

LL: lumbar lordosis
SS: sacral slope
BMI: body mass index
ASH: adjacent segment herniation
LDH: lumbar disc herniation
rLDHG: recurrent lumbar disc herniation group
ASHG: adjacent segment herniation group with or withot recurrence
ORs: odds ratios
PreLL: preoperative LL
PreSS: preoperative SS
PostLL: postoperative LL
PostSS: postoperative SS
one month after operation LL: one mLL
one month after operation SS: one mSS
